# Relationship between Onset of Sliding Behavior and Size of Droplet on Inclined Solid Substrate

**DOI:** 10.3390/mi13111849

**Published:** 2022-10-28

**Authors:** Yukihiro Yonemoto, Yosuke Fujii, Yoshiki Sugino, Tomoaki Kunugi

**Affiliations:** 1 Division of Industrial Fundamentals, Faculty of Advanced Science and Technology, Kumamoto University, 2-39-1, Kurokami, Chuo-ku, Kumamoto 860-8555, Japan; 2 Department of Mechanical and Mathematical Engineering, Kumamoto University, 2-39-1, Kurokami, Chuo-ku, Kumamoto 860-8555, Japan; 3 College of Energy Engineering, Zhejiang University, 38 Zheda Road, Hangzhou 310027, China

**Keywords:** wettability, sliding behavior, contact angle, surface tension, droplet

## Abstract

Whether a droplet slides or not on inclined solid surface is mainly influenced by a balance between the adhesion force at contact area and the gravitational force exerted on the droplet. Especially as the adhesion force is a key parameter for the determination of the sliding behavior of droplets. The adhesion force is mainly estimated by experimental observation for the sliding motion of the droplet. However, at present it is unknown whether the adhesion force is a constant value regardless of the droplet size or not. In the present study, focused on the onset for sliding of water-ethanol binary mixture droplets on inclined solid surface, experimental investigation on the sliding droplets is performed by considering the droplet volumes ranging from 7 to 600 μL in order to understand the effect of the size of the droplet on the adhesive property. The results are discussed using the existing analytical models. From the results, it is found that the adhesion force increases in the case of large droplet volume, while the force reaches constant value in the case of small droplet volume. This difference is related to the degree of the droplet shape deformation, which leads to a change in the contact angle. Finally, a simple empirical model for the adhesion force including the size effect is proposed.

## 1. Introduction

The control of liquid on solid substrate which is characterized by wettability is widely seen in industrial and chemical applications, such as coating, inkjet printing and spray cooling [1,2,3]. For example, in fuel cells [4], liquid water and droplet detachment from electrodes is a fundamental problem for water transport and its management. In a heat exchanger with dropwise condensation [5,6], the removal of droplets from solid surfaces is crucial problem for achieving higher heat transfer. In this kind of system, the dynamic motion of liquid on a solid surface is an important phenomenon where there are many factors related to wettability, adhesion force, flow field around the liquid, and inclination of solid substrate. 

There are many studies on the migration of liquid on solid substrates from experimental, numerical, and theoretical point of views. The migration behavior is one of the fundamental behaviors of dynamic wetting. However, in an actual situation, there are many unresolved problems which affect the migration of the liquid, such as internal fluid flow, solid surface condition, external forces, and the treatment of the dynamic contact angle. Yilbas et al. performed experimental and numerical works for the behavior of water droplets on an inclined hydrophobic surface. In their study, rolling behavior was investigated considering the fluid motion in the droplet with PIV (particle image velocimetry) technique. The result reveals the relationship between the fluid velocity in the droplet and the droplet volume [7]. Lv et al. considered the sliding behavior of water droplets on rough surfaces experimentally and analytically [8]. In their study, the effect of the surface microstructure (pillar-structured surface) on the onset of the sliding motion of droplet was discussed and the model for the prediction of the critical sliding angle on rough surface was proposed. Li et al. investigated water droplet detachment characteristics under different gas diffusion layer surfaces in proton exchange membrane fuel cell (PEMFC) experimentally and analytically [9]. In the experiment, the droplet detachment behaviors under air flow rates in the channel were observed. They revealed that the contact angle hysteresis, which is the difference between the advancing and receding contact angles, exhibits the linear relation with respect to the gas flow rate and the contact angle hysteresis of the droplet at the instant of the detachment decreases with the increase of the gas flow rate. In addition to the research mentioned above, there have been many studies focusing on droplet migration behavior, such as the droplet motion sheared by gas stream [10], the pinning characteristics of the droplet on heated or non-heated inclined surfaces [11,12], and modelling for the profile of the droplet on an inclined solid surface [13]. However, the migration behavior of the droplet is not fully understood.

The prediction of the onset for the droplet motion is mainly discussed on the basis of two models. Most popular models are developed by Furmidge [14] and Wolfram and Faust [15], which are mainly developed by considering the balance between external force and the surface tension based on the experimental observation where the sliding droplets on inclined solid surface are investigated. Concretely speaking, in the Wolfram and Faust’s (hereafter, called WF) model, the adhesion force per unit length of the contact line is defined as a single constant parameter. Thus, the WF model does not treat the contact angle. On the other hand, the adhesion force in the Furmidge’s model is evaluated using the surface tension and the contact angle hysteresis based on the concept of the Young’s equation. In a recent study [16], models for the retention force, which were related to the droplet migration behavior, were reviewed and introduced some types of Furmidge’s relations. The Furmidge type relation is widely used to understand the migration behavior instead of the WF model. In fact, a simple contact angle measurement of a static droplet is useful to understand and predict its surface conditions. It is mainly used as an indicator for the evaluation of the final determination for the surface preparation, and Furmidge’s relation is also used for the surface evaluation [17,18,19]. In the numerical simulation for the migration behavior [20,21], the values of the contact angle or the contact angle hysteresis where the droplet starts to slide are set as branch conditions. After the onset for the droplet motion, the treatment of the dynamic contact angle is needed, and the relationship between the contact line velocity and the dynamic contact angle is mainly discussed [22]. There are two approaches for the model: one is the molecular kinetic theory [23] and the other is the hydrodynamic theory [24,25]. The molecular kinetic theory is developed by considering the frequency of the random molecular displacements within the three phase zones, which are characterized by the activation free energy. The hydrodynamic theory is developed by considering the relationship between the viscous energy dissipation and the work achieved through the contact line motion. In both models, the contact angle is directly connected to the contact line velocity. However, in an actual situation, the relationship between the contact angle and the contact line velocity is not necessarily a one-to-one correspondence [26] and is not uniquely determined. Thus, at present, there are no versatile numerical models for the dynamic wetting behavior, and the modeling of the dynamic wetting behavior remains an unresolved problem [27].

Considered the dynamic motion of a droplet, the dynamic behavior is basically determined by the force balance among fluid motion, external force, surface tension and adhesion force at the solid–liquid interface, so the contact angle should be secondarily determined by this force balance. In particular, the adhesion force would work as a friction force and become an important factor for the determination of the dynamic contact angle during the dynamic motion. A recent study discusses the adhesion force evaluated by the Furmidge’s relation from a viewpoint of the concept of the friction coefficient [28]. If an inherent adhesion force between the liquid and solid surface exists, it would be a constant value regardless of whether a droplet size is large or not. However, it is unknown whether the adhesion force changes depending on the size of the droplet because most previous studies treat a relatively small droplet, such as several dozen μL or less [4,7,8,12,29]. Especially the WF and Furmidge’s models treat the same phenomena, so from an engineering point of view, it is important to know the difference in the adhesion forces between two models. Therefore, in the present study, the applicability of the WF and Furmidge’s models for the evaluation of adhesion force is considered in the wide range of the droplet volume, and the effect of the droplet size on the adhesion force is investigated. Finally, the relationship between the adhesion forces of two models are discussed and a simple model of the adhesion force, which includes the size effect, is proposed. 

## 2. Evaluation of Adhesiveness

### 2.1. Models for Adhesiveness of Droplet

In the present study, two existing models are used for the evaluation of the adhesiveness of droplets on a solid surface: the WF model [15] and the Furmidge’s model [14]. Two existing models mainly describe the phenomena for the sliding behavior of droplets on horizontal or inclined solid substrates and are briefly explained in the following section. 

### 2.2. Wolfram and Faust’s (WF) Model

In this model, the force balance between the adhesion force resulted from the wetted contact area and the gravitational force of droplet along the solid surface is mainly considered. In the model, the adhesion, *E*_w_, is defined as a force exerted on a unit length of the periphery of the contact area. From this concept, the following relation is derived.

(1) $$\rho_{l}V_{0}g\sin\alpha^{c}=\pi D_{0}E_{w}$$

In Equation (1), *ρ*_1_, *V*_0_, *g*, *α*^c^ and *D*_0_ represent the density of liquid, the initial volume of the droplet, gravitational acceleration, the critical inclined angle of the solid surface and the initial contact area diameter of the droplet, respectively. In this model, *E*_W_ is assumed to be constant. In addition, a contact angle is not considered. According to the concept of Young’s equation, the change in the contact angle indicates the change in the surface tension force acting on the contact line, even if the contact area does not change [30]. The contact angle is also an important factor for understanding droplet motion. Therefore, the applicability limit of Equation (1) is unknown if the size of the droplet increases, because the shape of the droplet is assumed as a part of sphere in this model. The evaluation procedure for *E*_w_ is as follows. By rewriting Equation (1), the relation sinα^c^ = π*E*_w_*D*_0_/(*ρ*_l_*gV*_0_) is obtained. Here, *E*_w_ is estimated by fitting the transformed relation to the experimental data where the linear relation of sin*α*_c_ and *D*_0_/*V*_0_ is assumed as sin*α*_c_ = *k D*_0_/*V*_0_. Here, *k* is a constant value. Therefore, *E*_w_ is obtained by the relation *E*_w_ = *ρ*_l_*gk*/*π*. 

### 2.3. Furmidge’s Model

In this model, the shape of the droplet (i.e., wetted contact area) is assumed to be a rectangle. The adhesion is evaluated using advancing and receding contact angles based on the concept for the Young’s equation. Then, the relationship is derived by considering the work performed by the gravity and the variation of the adhesion work in the sliding process. Nevertheless, the model is sufficiently able to capture the sliding droplet behavior and is applied to many migration phenomena by an addition of a pre-factor to the original Furmidge’s model [31,32,33]. The Furmidge’s model with the pre-factor *c_f_* is as follows:

(2) $$\rho_{l}V_{0}g\sin\alpha^{c}=c_{f}l_{\mathrm{width}}\sigma_{\mathrm{lg}}(\cos\theta_{R}-\cos\theta_{A})$$

In Equation (2), *c_f_*, *l*_width_, *σ*_lg_, *θ*_R_ and *θ*_A_ represent the pre-factor, the width of the contact area of the droplet, the surface tension between liquid and gas and the receding and advancing angles, respectively. Equation (2) reduces to the original Furmidge’s model when the pre-factor *c_f_* takes unity. In recent models, there are some expressions for the pre-factor *c_f_* where the Laplace pressure and the parameter for the pinning force are considered [16,34]. However, there is no consensus for the expression of the model. Therefore, the present study mainly focusses on the classical relations of the WF and the original Furmidge’s models, as mentioned in the next section, and the pre-factor is discussed in Section 4.3. In a previous study [35], it was revealed that the value of *l*_width_ is almost constant, which is the same as the initial droplet contact area diameter *D*_0_ until the onset of the droplet sliding. Therefore, in the present study, *l*_width_ in Equation (2) is treated as *D*_0_.

### 2.4. Alternative Evaluation for WF and Furmidge’s Models

A critical inclined angle in Equations (1) and (2) represents the onset of the droplet sliding motion. Therefore, if one applies the concept of Equation (1) to the Furmidge’s model, Equation (2) can be rewritten as follows:

(3) $$\sin\alpha^{c}=\frac{\sigma_{\mathrm{lg}}(\cos\theta_{R}-\cos\theta_{A})}{\rho_{l}g}\frac{l_{\mathrm{width}}}{V_{0}}$$

Note, the value of *l*_width_ can be treated as the initial droplet contact area diameter *D*_0_ until the onset of the sliding motion [35]. Thus, comparing Equation (3) with Equation (1), the value that corresponds to the adhesion force per unit of length defined as *E*_F_ can be derived as follows:

(4) $$E_{F}=\frac{\sigma_{\mathrm{lg}}(\cos\theta_{R}-\cos\theta_{A})}{\pi }$$

From Equation (4), it was found that *E*_F_ includes the geometrical parameter of the droplet, which is not considered in *E*_W_ of Equation (1), because *E*_W_ is estimated by fitting Equation (1) to the experimental data, assuming the linear relationship between sin*α*^c^ and *D*_0_/*V*_0_ as mentioned in the Section 2.2. This indicates that *E*_W_ may be the averaged value for the adhesion force in the wide range of the droplet volumes. Therefore, the following adhesion *E*_W_’ is evaluated in addition to Equation (4).

(5) $$E_{w}{}^{\prime }=\frac{\rho_{l}g}{\pi }\left(\sin\alpha^{c}(V_{0})\frac{V_{0}}{D_{0}}\right)$$

*E*_W_’ is evaluated using the experimental data for the onset of the droplet sliding motion in Equation (5). Finally, in the present study, three kinds of the adhesion force for *E*_W_, *E*_W_’ and *E*_F_ are evaluated. Note that the present study does not focus on the morphological effect of the solid surface, such as the surface roughness on the sliding behavior [19,36,37,38]. In order to consider such a problem, more detailed investigation would be needed, including the definition of the movement of the contact line, because the pinning effect on the contact line motion becomes significant.

## 3. Experiment

**Figure 1:**
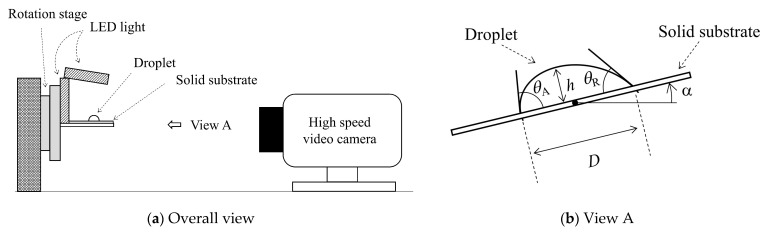
Schematic of (**a**) overall view of experimental apparatus and (**b**) view A in (**a**) where the geometrical parameters are defined.

**Figure 2:**
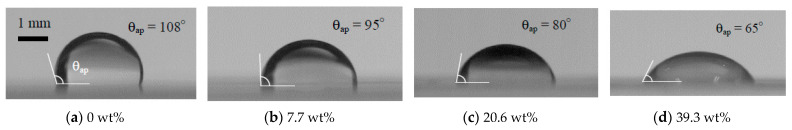
Droplet wettability of each liquid on silicone rubber (SR): (**a**) 0 wt% (0.072 Nm^−1^), (**b**) 7.7 wt% (0.051 Nm^−1^), (**c**) 20.6 wt% (0.038 Nm^−1^) and (**d**) 39.3 wt% (0.030 Nm^−1^). Droplet volume *V*_0_ is 10 μL.

Figure 1 shows the schematic of the experimental apparatus. As shown in Figure 1a, the apparatus mainly consists of the high-speed video camera (HX-5, NAC Image Technology, Ltd., Tokyo, Japan), the rotation stage and the LED light. The solid sample is set on the rotation stage. The droplet is deposited on the solid substrate as shown in Figure 1b. After the deposition, the solid substrate is rotated with a constant angular velocity ω = 0.5 deg sec^−1^. Then, the droplet motion during the rotation was captured with the high-speed video camera. The geometrical parameters, such as the contact area diameter *D*, height *h* and advancing (*θ*_A_) and receding (*θ*_R_) contact angles were measured. In the present study, silicone rubber (SR) was used as the solid substrate. The surface roughness of SR is Ra = 0.02 μm [39]. The SR substrate is a kind of low-surface-energy solid, which enables us to make a stable droplet shape on solid surface with high reproducibility, unlike a high-surface-energy solid, such as a metal [40]. Then, water–ethanol binary mixtures were used for the liquid. The four mixtures with different ethanol mass concentrations were used: 0.072 Nm^−1^ (0 wt%), 0.051 Nm^−1^ (7.7 wt%), 0.038 Nm^−1^ (20.6 wt%) and 0.030 Nm^−1^ (39.3 wt%). The droplet volumes ranged from 7 to 600 μL. More detailed information on the droplets is listed in Table 1. In this experiment, the temperature and humidity were in the ranges of 20.0–25.0 °C and 50.0–55.0%, respectively. Each experimental condition was performed three times. The contact angles of droplet were measured using commercial software (FAMAS; Kyowa Interface Science Co., Ltd., Saitama, Japan). Figure 2 shows the images of the droplet wettability on SR. The apparent contact angle decreases as the ethanol concentration increases. Here, the droplet volume is 10 μL in each liquid. 

## 4. Results and Discussion

### 4.1. Inclined Angle Dependency of Geometrical Parameters of Droplet

**Figure 3:**
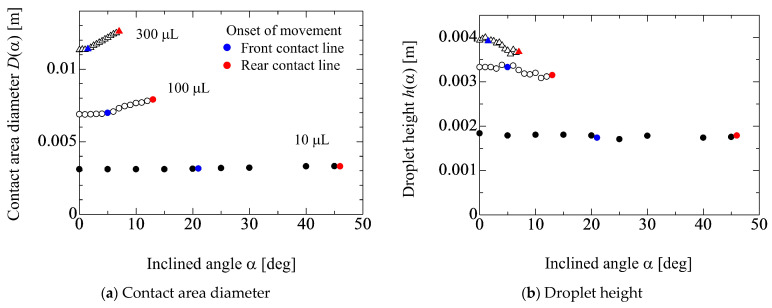
Relationship among contact area diameter *D*, height *h* of water droplet (0 wt%) and inclined angle *α* of solid substrate. The blue and red characters represent the front and rear contact lines movement, respectively.

Figure 3 shows the relationship between the inclined angle *α*, contact area diameter *D*(*α*) and the height *h*(*α*) of water droplets. The droplet volumes are 10, 100 and 300 μL. In this figure, blue and red points represent the onset of movement of the front and rear contact lines (FCL and RCL), respectively. From Figure 3a, it can be seen that *D*(*α*) increases after the front contact line starts to move. On the other hand, in Figure 3b, the droplet height *h*(*α*) decreases as *α* increases. The critical inclined angle where each contact line starts to move becomes small as the droplet volume increases. This obviously indicates that the gravitational force becomes dominant compared with the adhesion force. In fact, the gradient of d*D*(*α*)/d*α* increases and the relationship between *D*(*α*) and α exhibits linear as the droplet volume increases. 

**Figure 4:**
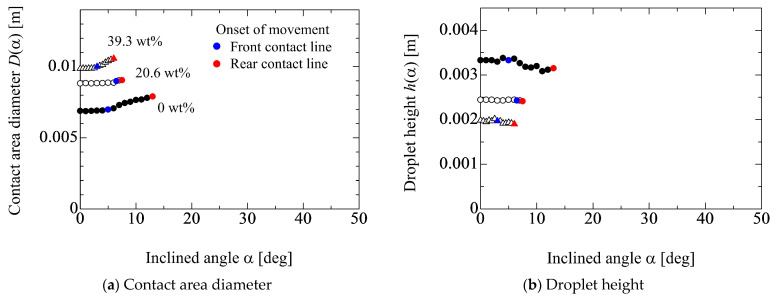
Ethanol concentration dependency on the behaviors for *D* and *h*. Droplet volume is 100 μL. The blue and red characters represent the front and rear contact lines movement, respectively.

**Figure 5:**
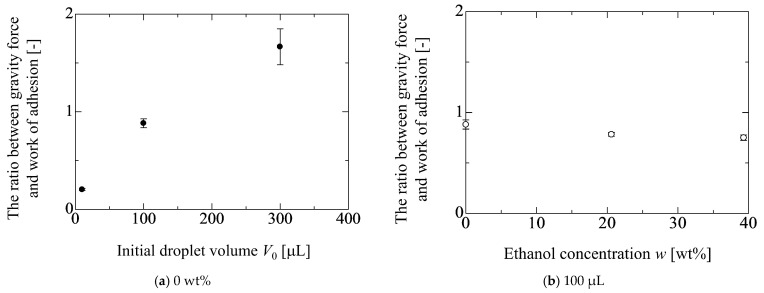
Ratio between gravity force per unit contact line and the work of adhesion. The ratio is calculated by *mg*/(π*D*_0_ σ_lg_ (1 + cos*θ*_0_).

Figure 4 shows the ethanol concentration dependency of the behaviors for *D*(*α*) and *h*(*α*). The droplet volume is 100 μL. The changes in *D*(*α*) and *h*(*α*) against *α* in Figure 4a,b are qualitatively the same, as shown in Figure 3. For example, in Figure 3a, the critical inclined angle for the movement of the front contact line becomes larger as *D*(*α*) increases (i.e., the droplet volume increases). In Figure 4a, the timing of the onset for the movement of the front contact line becomes fast as *D*(*α*) increases (i.e., the droplet wettability increases). However, the degree of the difference in the timing is quite different between the results in Figure 3 and Figure 4. This may be understood by considering the relationship between the gravity force and the work of adhesion based on the initial droplet condition. Concretely speaking, the ratio between the gravity force per unit contact line *mg*/(π*D*_0_) and the work of adhesion *σ*_lg_(1 + cos*θ*_0_) is considered for each liquid property. By this ratio, the behavior of the contact line is considered. Figure 5 shows the results for the ratio of two forces for each liquid property in Figure 3 and Figure 4. In Figure 5a, the ratio of two forces becomes large as the droplet volume increases, which indicates the gravity is dominant compared with the wettability. Therefore, the difference in the critical inclined angle for the movement of the front contact line (*α*^c^_FCL_) becomes large with respect to the droplet volume. On the other hand, from the result in Figure 5b, it is found that the wettability is dominant compared with the gravity force and the order of three values are similar. Thus, the differences in *α*^c^_FCL_ among the three liquid properties are not so large. As to the difference between *α*^c^_FCL_ and *α*^c^_RCL_ (the critical inclined angle for the movement of the rear contact line), the degree of the deformation for the droplet shape may be related. Concretely speaking, from the results in Figure 4b, the change in the droplet height between the substrate inclined angles at *α*^c^_RCL_ and *α*^c^_FCL_ in the water case is larger than that in the cases of 20.6 and 39.3 wt%. The deformable case, such as water, easily elongates the contact area diameter. Therefore, in the non-deformable cases, such as 20.6 and 39.3 wt%, the difference between *α*^c^_RCL_ and *α*^c^_FCL_ is not so large compared with water. In fact, in Figure 4, the averaged values of the difference between *α*^c^_RCL_ and *α*^c^_FCL_ for 0, 20.6 and 39.3 wt% are 8.7, 2.5 and 2.3 deg, respectively. 

**Figure 6:**
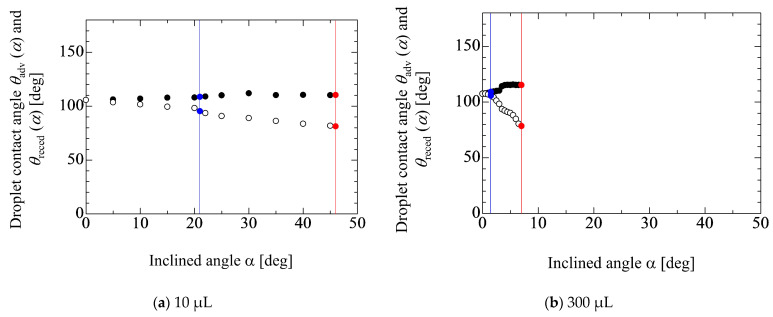
Change in advancing and receding the contact angles of water droplets during the inclination of the solid substrate. The blue and red solid lines represent the front and rear contact lines movement, respectively.

**Figure 7:**
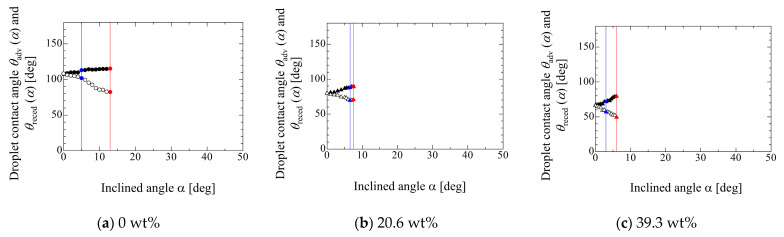
Effect of the liquid property on the changes in the contact angles. The blue and red solid lines represent the front and rear contact lines movement, respectively.

Figure 6 shows the changes in the advancing and receding contact angles during the inclination of the solid substrate. The results for water droplets of 10 and 300 μL are depicted in this figure. From these results, the gradients of |d*θ*(*α*)/d*α*| between the advancing and receding contact angles after the movement of the front contact line are different from each other. This may result from the fact that water exhibits a hydrophobic condition against the SR substrate, which means that the contact line is basically hard to move. In addition, the front contact line moves towards the dry surface and the rear contact line moves towards the wet surface. These conditions may induce the difference in the gradients. Figure 7 shows the effect of the liquid property on the changes in the contact angles. The mass concentrations of ethanol are 0 wt%, 20.6 wt% and 39.3 wt%. The droplet volumes are the same at 100 μL in each case. In the cases of 20.6 wt% and 39.3 wt%, the gradients of |d*θ*(*α*)/d*α*| between the advancing and receding contact angles are similar to each other. This may result from two cases that are relatively hydrophilic against the solid surface, which indicates that the contact line is easy to move. Therefore, it was thought that the difference in the surface conditions, such as wettability, in these cases does not strongly reflect to the differences in the gradients of |d*θ*(*α*)/d*α*|. 

### 4.2. Evaluation of Adhesion Forces

**Figure 8:**
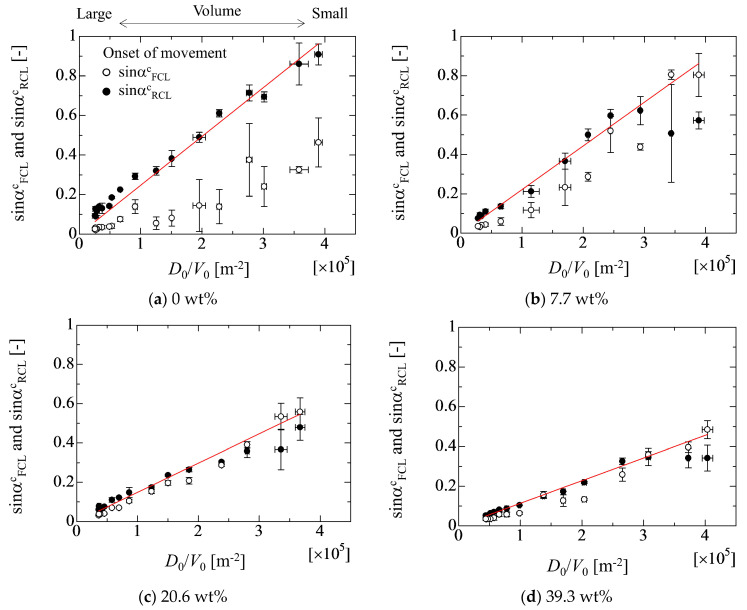
Relationship between critical inclined angles of sinα^c^_FCL_ and sinα^c^_RCL_ and *D*_0_/*V*_0_ in each liquid property.

Figure 8 shows the relationship between sin*α*^c^_FCL_ or sin*α*^c^_RCL_ and *D*_0_/*V*_0_ in each liquid property. The open and solid circles represent the critical inclined angles of sin*α*^c^_FCL_ and sin*α*^c^_RCL_, respectively. The red solid line represents the linear fitting by Equation (1) to the experimental data. The critical inclined angles of sin*α*^c^_FCL_ and sin*α*^c^_RCL_ indicate the points where the front and rear contact lines start to move, respectively. Equation (1) is applied to the condition where the droplet moves. For example, as shown in Figure 7a, if the front contact line moves at first where the rear contact line is pinned, after that, the rear contact line starts to move and slide, the open circle is below the solid one. Therefore, the red solid line is used for the fitting. This means that the linear fitting of Equation (1) for the experimental data includes both conditions of sinα^c^_FCL_ and sin*α*^c^_RCL_, as seen in Figure 8b–d. From the results of Figure 8, most data show that the front contact line moves before the rear contact line moves. In addition, the differences between the points of front and rear contact line becomes small as the ethanol concentration increases. By fitting Equation (1) to the experimental data shown in Figure 8, the adhesion force of *E*_W_ and *E*_W_’ can be evaluated for each liquid property. *E*_F_ in Equation (4) can be evaluated based on the experimental data for the contact angles of droplet, as shown in Figure 6 and Figure 7. 

**Figure 9:**
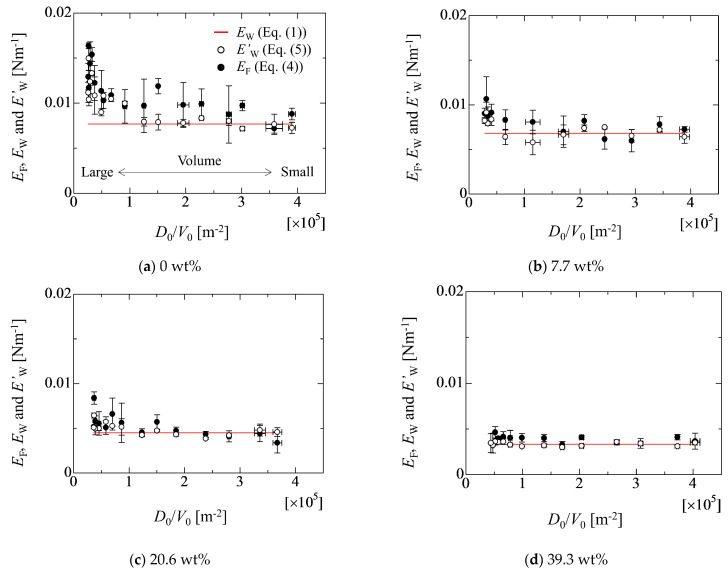
Relationship between adhesion force and *D*_0_/*V*_0_ in each liquid property.

Figure 9 shows the droplet size dependency of the adhesion forces in each liquid property. The red solid line indicates the adhesion force of *E*_W_ evaluated by the linear fitting approach. Here, the estimated values of *E*_W_ are 7.7 × 10^−3^, 6.8 × 10^−3^, 4.5× 10^−3^ and 3.3 × 10^−3^ Nm^−1^ for 0 wt%, 7.7 wt%, 20.9 wt% and 39.3 wt%, respectively. The open and solid circles represent the adhesion forces of *E*_F_ and *E*_W_’, respectively. The results indicate that the values of *E*_W_’ and *E*_F_ in the case of 0 wt% deviate from the value of *E*_W_ as the droplet volume increases. In Figure 9a, the experimental data of *E*_W_’ and *E*_F_ largely deviate from the linear line of *E*_W_ as the droplet volume increases. On the other hand, the deviation of *E*_W_’ and *E*_F_ from *E*_W_ gradually decreases as the ethanol concentration increases. In the case of 39.3 wt%, the values of *E*_W_’ and *E*_F_ almost coincide with *E*_W_. This volume effect on the adhesion forces may result from the deformation of the droplet shape due to the gravity. The cases from the case (b) to case (d) are basically hydrophilic condition against the SR solid substrate. This means that the droplet height is low and the center of gravity is close to the solid surface. On the other hand, case (a) displays hydrophobic conditions. The center of gravity is far from the solid surface compared with the hydrophilic one. Therefore, the droplet shape easily deforms due to the gravity force in the hydrophobic case. 

**Figure 10:**
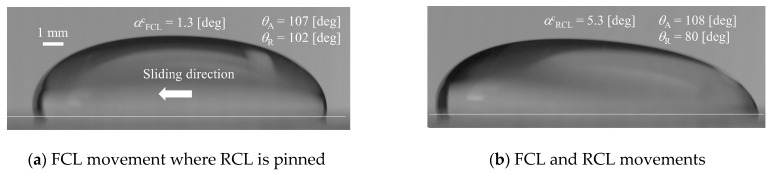
Images for onset of each contact line (CL) of the water droplet (550 μL).

**Figure 11:**
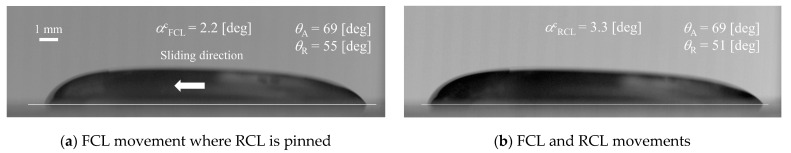
Images for onset of each contact line (CL) of the 39.3 wt% droplet (400 μL).

**Figure 12:**
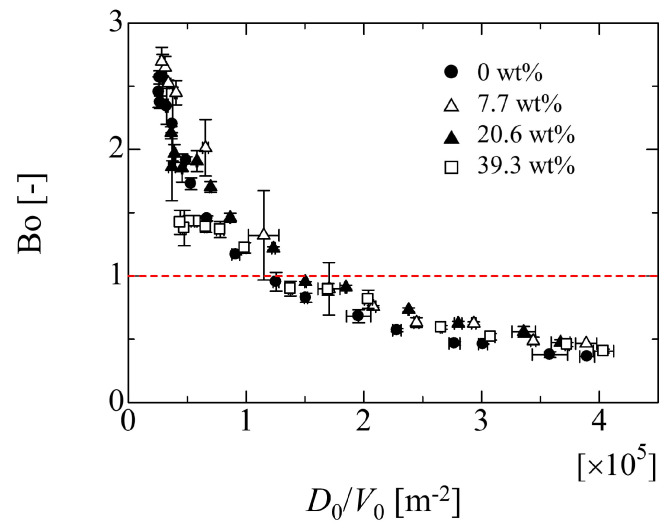
Relationship between Bo number of initial droplet and *D*_0_/*V*_0_.

Figure 10a,b represents the images when the front and rear contact line starts to move, respectively. From Figure 10a, it can be seen that the advancing contact angle almost takes the same value as the receding one. However, in Figure 10b, the receding contact angle is smaller than the advancing one, which implies the deformation of the droplet shape. On the other hand, in the case of 39.3 wt%, as shown in Figure 11, the advancing and receding contact angles take similar values in both cases of Figure 11a,b. This means that the droplet deformation almost does not arise in the case of high ethanol concentration. From Figure 9, in addition to the result in Figure 8, at least, it can be seen that the effect of the droplet size on the adhesion force is not so significant if the value of *D*_0_/*V*_0_ is larger than 1.5 × 10^5^ m^−2^. Here, Figure 12 shows the relationship between the bond number (Bo = *ρ*_l_*gh*_0_^2^/*σ*_lg_) evaluated by the initial droplet information and *D*_0_/*V*_0_ in each liquid property. In this figure, this criterion physically means the boundary where the Bo is less than unity depicted by the red dashed line.

### 4.3. Effect of Droplet Size on Adhesion Force

From the discussion in the previous section, it can be seen that the droplet size effect on the adhesion force *E*_F_ becomes large as the droplet volume increases, as shown in Figure 9. Thus, by focusing on *E*_W_ and *E*_F_, the effect of the droplet size on the adhesion force can be considered, as the ratio between *E*_W_, which is constant, and *E*_F_: *E*_W_/*E*_F_. Note that, in this section, it is assumed that the inherent adhesion force between the liquid and solid is expressed by *E*_W_, which is obtained by the linear relationship between sinα_c_ and *D*_0_/*V*_0_.

Since the result for Bo in Figure 12 exhibits a similar trend in Figure 9, the ratio *E*_W_/*E*_F_ may be related to Bo. In particular, from the expression of Equation (2), the ratio *E*_W_/*E*_F_ indicates a pre-factor *c_f_* and means the factor which corrects *E*_F_ to *E*_W_ if the constant *E*_W_ is thought as the adhesion forces between liquid and solid. From the results in Figure 9 and Figure 12, a simple relationship can be deduced as follows:

(6) $$\frac{E_{w}}{E_{F}}=aBo+b$$

**Figure 13:**
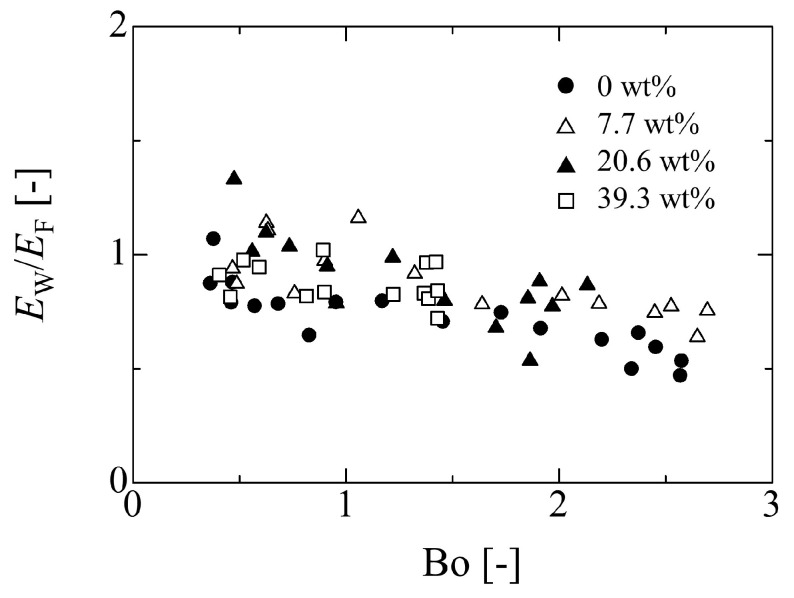
Relationship between Bo number and the ratio of adhesion force *E*_W_ and *E*_F_.

Here, from the result in Figure 9, the *E*_F_ approaches *E*_W_ as the droplet size becomes small (increase of *D*_0_/*V*_0_). This indicates one limit condition in Equation (6) that *b* is unity because the size effect on the adhesion force becomes small and *E*_W_ ≈ *E*_F_ when Bo→0. Figure 13 shows the relationship between the ratio *E*_W_/*E*_F_ and Bo in each liquid case. The trend in the figure exhibits a relatively linear relationship between *E*_W_/*E*_F_ and Bo. By fitting Equation (6) to the experimental data in Figure 13, the value *a* is estimated as −1.31 × 10^−1^ ± 0.42 × 10^−1^. 

**Figure 14:**
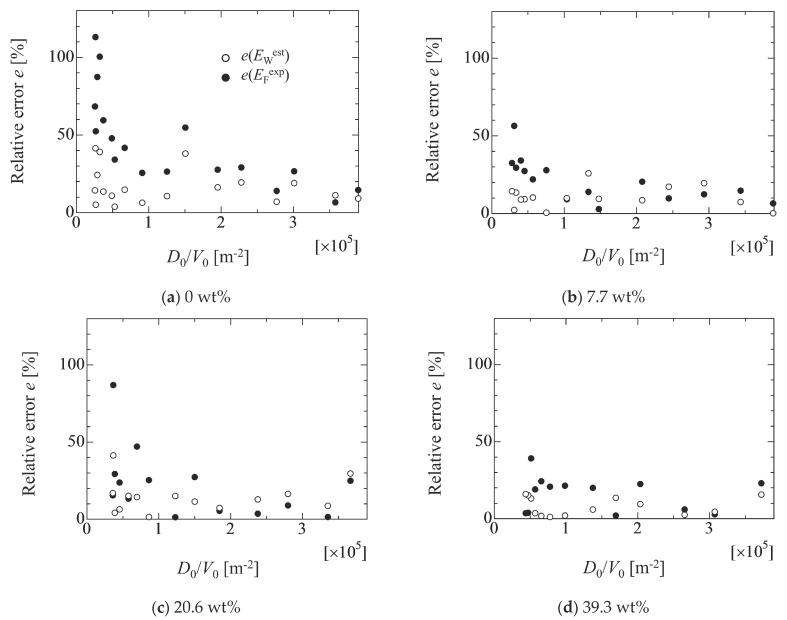
Relative errors of *e*(*E*_W_^est^) and *e*(*E*_F_^exp^) in each liquid case. The values of *e*(*E*_W_^est^) and *e*(*E*_F_^exp^) are calculated by Equation (7) as 100 × |(*E*_W_ − *E*_W_^est^)/*E*_W_| and 100 × |(*E*_W_ − *E*_W_^exp^)/*E*_W_|, respectively. *E*_W_, *E*_F_^exp^ and *E*_W_^est^ are estimated by Equations (1), (4) and (6), respectively.

The relative errors among the adhesion forces of *E*_W_, *E*_F_^exp^ and *E*_W_^est^ (Equation (6)) are evaluated in Figure 14. Here, the relative error is calculated by the following relation.

(7) $$e(E)=100\times \left|\frac{E_{W}-E}{E_{W}}\right|$$

In the figure, the values of *E*_F_^exp^ and *E*_W_^est^ are substituted into *E* in Equation (7), and the white and black circles represent the relative error of *e*(*E*_W_^est^) and *e*(*E*_F_^exp^), respectively. The estimated values of *E*_W_^est^ by Equation (6) shows relatively good agreement with the linear fitting value of *E*_W_; for example, the relative error for *E*_F_^exp^ becomes large as the droplet volume increases in each liquid case. On the other hand, the relative errors for *E*_W_^est^ are smaller than that that for *E*_F_^exp^. This indicates that the size effect of the droplet on the adhesion force can be well correlated by the simple linear relation of Equation (6). In fact, it is reported that the pre-factor *c_f_* in Equation (2) is related to the size of the droplet [32,33]. However, there is a large discrepancy for the water case in the case of the large droplet. In the water case, the deformation of the droplet is larger than that of other cases. This might relate to the lack of the consideration for the physical conditions, such as a force balance at the contact line and the effect of the droplet surface shape on the adhesion force. Concretely speaking, the models mentioned in the Section 2 are mainly the retention force in the horizontal direction at the contact line. However, forces such as the vertical force at the contact line and the Laplace pressure of the droplet surface are also important factors to determine the droplet conditions. Therefore, such factors must be considered in Equation (6) from a comprehensive point of view in the future.

## 5. Conclusions

The sliding behavior of water–ethanol binary mixture liquids on the silicone rubber was experimentally investigated. The adhesion forces between the liquid and solid surface were evaluated based on the existing models. In particular, the size effect on the adhesion forces were considered by setting the wide range of droplet volumes. 

From the results, the critical inclined angle where the front contact line (FCL) starts to move is smaller than that of the rear contact line (RCL) in the case of 0 wt%. However, as the ethanol concentration increases, two values of the critical inclined angles take similar one. Although it was found that the relationship between the critical inclined angle where the droplet starts to move and the *D*_0_/*V*_0_ exhibits almost a linear relation in the high ethanol concentration case, the data for large droplets in the case of 0 wt% deviate from the linear relation. In fact, the adhesion force *E*_F_ evaluated using the droplet contact angles (Equation (4)) exhibits larger value than that of the adhesion force of *E*_W_ evaluated by a linear fitting approach (Equation (1)). This may result from the deformation of the droplet shape where the deformation of the hydrophobic case is larger than that of the hydrophilic case. This means that the adhesion forces will be influenced by the droplet volume (deformation) if the existing models are used for the evaluation of the adhesion force. From the present study, at least, it was found that the effect of the droplet size on the adhesion force is not so large if the value of *D*_0_/*V*_0_ is larger than 1.5 × 10^5^ m^−2^. This criterion is the boundary where the Bo of the initial droplet is less than unity. This would become one of the judgement criteria for the appropriate droplet volume for the evaluation of the adhesion force. Furthermore, to consider the effect of the droplet deformation on the adhesion force, the relationship between *E*_W_/*E*_F_ and Bo is considered. The result indicates that the *E*_W_/*E*_F_ exhibits the good linearity with respect to Bo, which means that the droplet deformation can mainly be considered by the Bo. However, for increased understanding of the larger deformation of the droplet, further detailed investigation is needed by considering the vertical force relation and the Laplace force exerted on the droplet because the relationship used in the present work is only the lateral force relation on the solid surface. 

## Figures and Tables

**Table 1:** Droplet volumes, initial contact area diameter and droplet height in each liquid used in sliding experiment.

Liquids [wt%]	Volumes [μL]	Initial Contact Are Diameter *D*_0_ [m]	Initial Droplet Height *h*_0_ [m]
0	7–600	2.7 × 10^−3^–15.5 × 10^−3^	1.6 × 10^−3^–4.3 × 10^−3^
7.7	8–600	3.1 × 10^−3^–16.9 × 10^−3^	1.6 × 10^−3^–3.7 × 10^−3^
20.6	10–500	3.7 × 10^−3^–18.5 × 10^−3^	1.4 × 10^−3^–2.7 × 10^−3^
39.3	10–400	4.0 × 10^−3^–17.6 × 10^−3^	1.2 × 10^−3^–2.2 × 10^−3^

## Data Availability

The data presented in this study are available in article.

## Nomenclature

The parameters used in the present paper are listed below.

**Symbols**	
Bo	Bond number [-]
*a*, *b*	Parameters in Equation (6) [-]
*c_f_*	Pre-factor in Furmidge’s relation [-]
*D*	Contact area diameter of droplet [m]
*E*	Adhesion force [Nm^−1^]
*e*	Relative error [%]
*g*	Gravitational acceleration [m s^−2^]
*h*	Droplet height [m]
*k*	Gradient of linear fitting relation for Wolfram and Faust’s model [m^2^]
*l*_width_	Width of the contact area of droplet [m]’
*V*	Droplet volume [m^3^]
**Greek Symbols**	
*α*	Inclined angle of solid substrate [deg]
*θ*	Contact angle [deg]
*ρ*	Density [kg m^−3^]
*σ*	Surface tension [N m^−1^]
ω	Angular velocity of rotation for solid substrate [deg s^−1^]
**Superscripts**	
c	Critical
est	Estimation
exp	Experiment
**Subscripts**	
0	Initial condition
A	Advancing
ap	Apparent
F	Furmidge’s model
FCL	Front contact line
*l*	Liquid
*lg*	Liquid–gas interface
R	Receding
RCL	Rear contact line
W	Wolfram model
